# Demethylation of the miR-146a promoter by 5-Aza-2’-deoxycytidine correlates with delayed progression of castration-resistant prostate cancer

**DOI:** 10.1186/1471-2407-14-308

**Published:** 2014-05-02

**Authors:** Xiaolu Wang, Haitao Gao, Lixin Ren, Junfei Gu, Yanping Zhang, Yong Zhang

**Affiliations:** 1 Department of Urology, The Second Hospital of Hebei Medical University, No. 215 Hepingxi Road, Shijiazhuang 050000, China

**Keywords:** Prostate cancer, 5-Aza-2’-deoxycytidine (5-Aza-CdR), DNA methyltransferases (DNMTs), miR-146a, Castration

## Abstract

**Background:**

Androgen deprivation therapy is the primary strategy for the treatment of advanced prostate cancer; however, after an initial regression, most patients will inevitably develop a fatal androgen-independent tumor. Therefore, understanding the mechanisms of the transition to androgen independence prostate cancer is critical to identify new ways to treat older patients who are ineligible for conventional chemotherapy.

**Methods:**

The effects of 5-Aza-2’-deoxycytidine (5-Aza-CdR) on the viability and the apoptosis of the androgen-dependent (LNCaP) and androgen-independent (PC3) cell lines were examined by MTS assay and western blot analysis for the activation of caspase-3. The subcutaneous LNCaP xenografts were established in a nude mice model. MiR-146a and DNMTs expressions were analyzed by qRT-PCR and DNA methylation rates of LINE-1 were measured by COBRA-IRS to determine the global DNA methylation levels. The methylation levels of miR-146a promoter region in the different groups were quantified by the bisulfite sequencing PCR (BSP) assay.

**Results:**

We validated that 5-Aza-CdR induced cell death and increased miR-146a expression in both LNCaP and PC3 cells. Notably, the expression of miR-146a in LNCaP cells was much higher than in PC3 cells. MiR-146a inhibitor was shown to suppress apoptosis in 5-Aza-CdR-treated cells. In a castrate mouse LNCaP xenograft model, 5-Aza-CdR significantly suppressed the tumors growth and also inhibited prostate cancer progression. Meanwhile, miR-146a expression was significantly enhanced in the tumor xenografts of 5-Aza-CdR-treated mice and the androgen-dependent but not the androgen-independent stage of castrated mice. In particular, the expression of miR-146a was significantly augmented in both stages of the combined treatment (castration and 5-Aza-CdR). Additionally, the methylation percentage of the two CpG sites (−444 bp and −433 bp), which were around the NF-κB binding site at miR-146a promoter, showed the lowest methylation levels among all CpG sites in the combined treatment tumors of both stages.

**Conclusion:**

Up-regulating miR-146a expression via the hypomethylation of the miR-146a promoter by 5-Aza-CdR was correlated with delayed progression of castration-resistant prostate cancers. Moreover, site-specific DNA methylation may play an important role in miR-146a expression in androgen-dependent prostate cancer progression to androgen-independent prostate cancer and therefore provides a potentially useful biomarker for assessing drug efficacy in prostate cancer.

## Background

Prostate cancer is the most common malignant tumor in men in many industrialized nations and is the second highest cause of cancer mortality [1]. As prostate cancer relies on androgens for its maintenance and progression, targeting androgens offers a therapeutic opportunity to halt or delay the progression of prostate cancer. Androgen-deprivation therapies, including castration (orchiectomy, the surgical removal of the testicles) and pharmacological control, are the mainstay for the management of advanced prostate cancer, reducing symptoms in approximately 70–80% of patients. Regardless, most tumors relapse within two years to an incurable hormone-independent state, in which tumor cells are scarcely responsive to even high concentrations of chemotherapeutic agents or radiotherapy [2]. The regulatory mechanisms that cause this transition remain largely unknown, and no effective therapy for androgen-independent prostate cancer (AIPC) has been developed to date.

DNA methylation is an important regulator of gene transcription and plays an important role in the process of tumorigenesis and the development of prostate cancer [3,4]. 5-Aza-2’-deoxycytidine (5-Aza-CdR), a nucleoside analog inhibitor of DNA methyltransferase (DNMT), has been used to reverse methylation and reactivate the expression of silenced genes [5]. 5-Aza-CdR was able to suppress the growth of various tumors in vitro, animal models, and clinical trials including prostate cancer [6-9], hematopoietic malignancies [10,11], and lung carcinoma [12]. In vitro studies investigating the effects of 5-aza-CdR in prostate cancer cell lines demonstrated that a low-dose 5-aza-CdR treatment regimen given daily completely inhibited cell proliferation and induced cell death in LNCaP and PC3 cell lines [13]. Moreover, several studies have been conducted to examine the synergistic effects of 5-Aza-CdR and chemotherapeutic agents against tumor cells. A combination of 5-Aza-CdR and cisplatin showed synergy in triggering the apoptotic death of PC3 cells [14], and the combined treatment of castration and 5-Aza-CdR was more effective than either treatment alone according to tissue histology, significantly prolonging survival in a transgenic adenocarcinoma mouse model of prostate cancer [9]. Nevertheless, it remains unknown whether 5-Aza-CdR can delay the progression of castration-resistant prostate cancer, and little is known about the genes silenced in progressed prostate cancer cells that are reactivate by 5-Aza-CdR.

MicroRNAs (miRNAs) are a class of short (between 19 and 25 nucleotides) noncoding RNAs that negatively regulate gene expression via complementary binding to target messenger RNA (mRNA) [15], thereby impairing the translation of the mRNA or marking it for early degradation. Dysregulated miRNA expression plays an important role in the process of tumorigenesis and development of androgen-independent tumors after castration in prostate cancer patients [16]. A recent study reported that the excessive expression of miR-146a was exclusively found in prostate cancer LNCaP and PC3 cell lines [17]. Transfection of miR-146a into a cell line was found to significantly suppress the expression of ROCK1, consequently markedly reducing cell proliferation, invasion, and metastasis to human bone marrow endothelial cell monolayers [17,18], indicating that miR-146a may function as a tumor-suppressor gene in the transformation of AIPC and metastasis in prostate cancer.

In the present study, we found that 5-Aza-CdR could inhibit the cell viability of either androgen-dependent LNCaP cells or androgen-independent PC3 cells through the up-regulation of miR-146a expression. To evaluate the effect of 5-Aza-CdR on the progression of AIPC in vivo, we established subcutaneous LNCaP xenografts in castrated male mice. As expected, 5-Aza-CdR delayed the progression of castration-resistant tumors. The methylation levels of the global genome and miR-146a promoter were decreased after 5-Aza-CdR treatment, which resulted in the increased expression of miR-146a, suggesting that castration in combination with 5-Aza-CdR may be a new therapeutic option for both androgen-dependent and -independent prostate cancer.

## Methods

### Cell culture and treatment

LNCaP and PC3 human prostate carcinoma cells (American Type Culture Collection, ATCC) were maintained in RPMI 1640 supplemented with 10% fetal bovine serum (FBS), 100 U/ml penicillin, and 100 pg/ml streptomycin (Gibco, NY, USA). 5-Aza-CdR (Sigma-Aldrich, St. Louis, Mo, USA) was dissolved in dimethylsulfoxide (DMSO). Fresh medium containing 5-Aza-CdR was replaced every 24 h. MiR-146a inhibitors (single-stranded chemically modified oligonucleotides; Life Technologies Corporation, Shanghai, China) were used for the inhibition of miR-146a expression in LNCaP and PC3 cells. Negative control inhibitors (ctrl inhibitors) were transfected as matched controls. Cells were transfected with RNAs using INTERFERin (Polyplus-Transfection SA, Illkirch, France) according to the manufacturer’s instructions.

### Cell viability assays

LNCaP and PC3 cells were seeded in 96-well culture plates with 100 μl of growth medium. Following 5-Aza-CdR or miR-146a inhibitor treatment, MTS cell viability assays were performed according to the manufacturer’s instructions.

### LNCaP xenograft model studies

Male BALB/c nude mice at 4–6 weeks age (18–20 g) were obtained from the Institute of Zoological Sciences, Chinese Academy of Medical Sciences in Beijing. For the tumor growth studies, 5 × 10^6^ LNCaP cells in 0.1 ml suspension were mixed with 0.1 ml of Matrigel (Collaborative Research Inc., Bedford, MA, USA) and inoculated into the right dorsal flanks of the mice. When the tumor volumes reached approximately 250 mm^3^, the animals were randomly divided into four groups (n = 12). One was administered thrice weekly on consecutive days intraperitoneal (i.p.) injections of 0.25 mg/kg 5-Aza-CdR dissolved in PBS. Two groups underwent bilateral orchiectomy (castration) under metofane (Pitman-Moore, Washington Crossing, NJ) anesthesia. Three days after surgery, these two groups were injected with either 5-Aza-CdR or PBS thrice weekly i.p. The control mice were testis-intact animals, with injections of PBS. The tumor volume was measured with calipers twice weekly for length and width; the volume was then calculated by the formula: [(length × width^2^)/2]. The animals of each group were euthanized on the 14th and 35th day post-treatment (n = 6 per group at either time point), respectively. All animal experiments were approved by the Committee of Use and Care of Animals, Chinese Academy of Medical Sciences.

### Measurement of miRNA and mRNA expression

The small and total RNA fractions were isolated isolated from cells and tumor tissue using the miRVana miRNA Isolation Kit (Ambion Inc, Austin, TX, USA) according to the manufacturer’s instructions. MiRNA was reverse-transcribed using the TaqMan microRNA Reverse Transcription Kit (Applied Biosystems, Foster City, CA, USA) in a reaction mixture containing an miR-specific stem-loop reverse transcription (RT) primer. The Taqman miRNA assay system (Applied Biosystems, Foster City, CA) was used according to the manufacturer’s instructions to quantitatively detect the expression of mature miRNAs was performed using the TaqMan miRNA Assay Kit (Applied Biosystems) containing TaqMan primers in a universal PCR master mix. CDNA was synthesised by reversetranscription using ReverTra Ace (Toyobo, Osaka, Japan). The SYBR Green PCR Master Mix (Toyobo, Osaka, Japan) was used to analyze mRNA expression. Quantification of miRNAs and mRNA by qRT-PCR was performed using an ABI 7300HT thermocycler (Applied Biosystems) at 95°C for 10 min, followed by 40 cycles of 95°C for 15 s and 60°C for 1 min. Reactions were performed in triplicate with human U6 or GAPDH as an internal control. The primer sequences for real-time quantitative PCR (qRT-PCR) are shown in Table 1. Fluorescent signals were normalized to an internal reference, and the threshold cycle was set within the exponential phase of PCR. The tumor tissue from the control mice of the first analysis was used to the calibrator samples. The relative gene expression was calculated by comparing cycles for each target PCR. Cycle threshold values were converted to relative gene expression levels using the 2^-ΔΔCT^ method.

**Table 1:** Primers for PCR and BSP

**Gene**	**Primer sequence**
DNMT1	F: TACCTGGACGACCCTGACCTC
	R: CGTTGGCATCAAAGATGGACA
DNMT3a	F: TATTGATGAGCGCACAAGAGAGC
	R: GGGTGTTCCAGGGTAACATTGAG
DNMT3b	F: GGCAAGTTCTCCGAGGTCTCTG
	R: TGGTACATGGCTTTTCGATAGGA
GAPDH	F: CGACCACTTTGTCAAGCTCA
	R: AGGGGTCTACATGGCAACTG
LINE1	F: CCGTAAGGGGTTAGGGAGTTTTT
	R: RTAAAACCCTCCRAACCAAATATAAA
HmiR-146a promoter	F: AGGGAGTTTTTTGTTTGATTTTTTTT
	R: CTATCCACCCTTTAACATACCTTCC

F, forward primer; R, reverse primer.

### Western blot analysis

Cells were lysed for 30 min at 4°C in PBS with 1% NP-40 and protease inhibitor cocktail tablets (Roche, Mannheim, Germany). Protein concentration was assayed by using bicinchoninic acid (Pierce, Rockford, IL). Total proteins were separated by electrophoresis on 12% SDS-polyacrylamide gels and 4% polyacrylamide gels and transferred to nitrocellulose. Immunodetection of caspase 3 and GAPDH was carried out by using anti-caspase 3 antibody at a dilution of 1:400 and anti-GAPDH (Cell Signaling Technology, Inc., Beverly, MA, USA). Specific proteins were visualized with enhanced chemiluminescence reagents (Hyperfilm ECL; Amersham Biosciences, Buckinghamshire, England) followed by exposure to X-ray film for 1 to 3 min.

### ELISA assay

Blood samples were collected by tail vein incision, and the serum was stored at −20°C until assayed. The free prostate-specific antigen (FPSA) in mouse serum was measured using ELISA kits (R&D Systems, USA) according to the manufacturer’s protocol.

### COBRA assay

The bisulfite modification of genomic DNA was performed using the CpGenome Turbo Bisulfite Modification Kit (Billerica, MA, USA), and the methylation analysis of LINE-1 repetitive elements was performed initially using the COBRA assay. The primer sequences that correspond to the nucleotides in the regulatory region of the LINE-1sequence are shown in Table 1. The PCR reactions consisted of 35 cycles of 95°C for 1 min, 53°C for 1 min, and 72°C for 1 min. The PCR products were subsequently digested with *TaqI* (MBI Fermentas) and *TasI* (MBI Fermentas) in TE buffer3 (Biolab) at 65°C overnight and then separated by electrophoresis on polyacrylamide gels. The gel was stained by silver staining. The methylated bands of LINE-1 (*Taq*I positive) yielded two 80-bp DNA fragments, whereas the unmethylated amplicons (*Tas*I positive) yielded 63- and 97-bp fragments. The methylation levels were calculated as the intensity of methylated bands divided by the sum of the methylated and unmethylated bands. Each COBRA assay was performed two to four times.

### Quantification of the methylation levels of the miR-146a promoter

The miR-146a promoter regions essential for basal transcriptional activity occur between nt −442 and nt +5 and include 11 CpG sites. The 447-bp region was amplified by bisulfite specific PCR (BSP) using primers designed with the MethPrimer software (Table 1). The PCR reaction contained modified genomic DNA and was overlaid with mineral oil to form a vapor barrier. The cycling conditions consisted of an initial denaturation at 96 ºC for 5 min after which 0.5 μl reverse primer (10 μM) was added to the PCR mixture, followed by 2 cycles of 96°C for 1 min, 58°C for 2 min, and 72°C for 2 min. When the temperature again rose to 96°C, which was at the beginning of the next phase of 8 cycles (96°C for 1 min, 58°C for 2 min, and 72°C for 2 min), 0.5 μl forward primer was added to the mixture. Then, 30 cycles of 96°C for 30 s, 55°C for 45 s, and 72°C for 45 s were performed, followed by a final extension of 72 ºC for 7 min. The PCR products were sequenced using a DNA sequencer (ABI PRISM 3730, Foster City, CA, USA), and the methylation levels were measured according to our previously reported method [19].

### Statistical analysis

All the data were obtained from at least three independent experiments. The data were expressed as the mean values ± SD and compared between two groups using Student’s *t*-test. The statistical significance was defined as a P value less than 0.05.

## Results

### 5-Aza-CdR-induced miR-146a expression promoted apoptosis in LNCaP and PC3 cells

**Figure 1:**
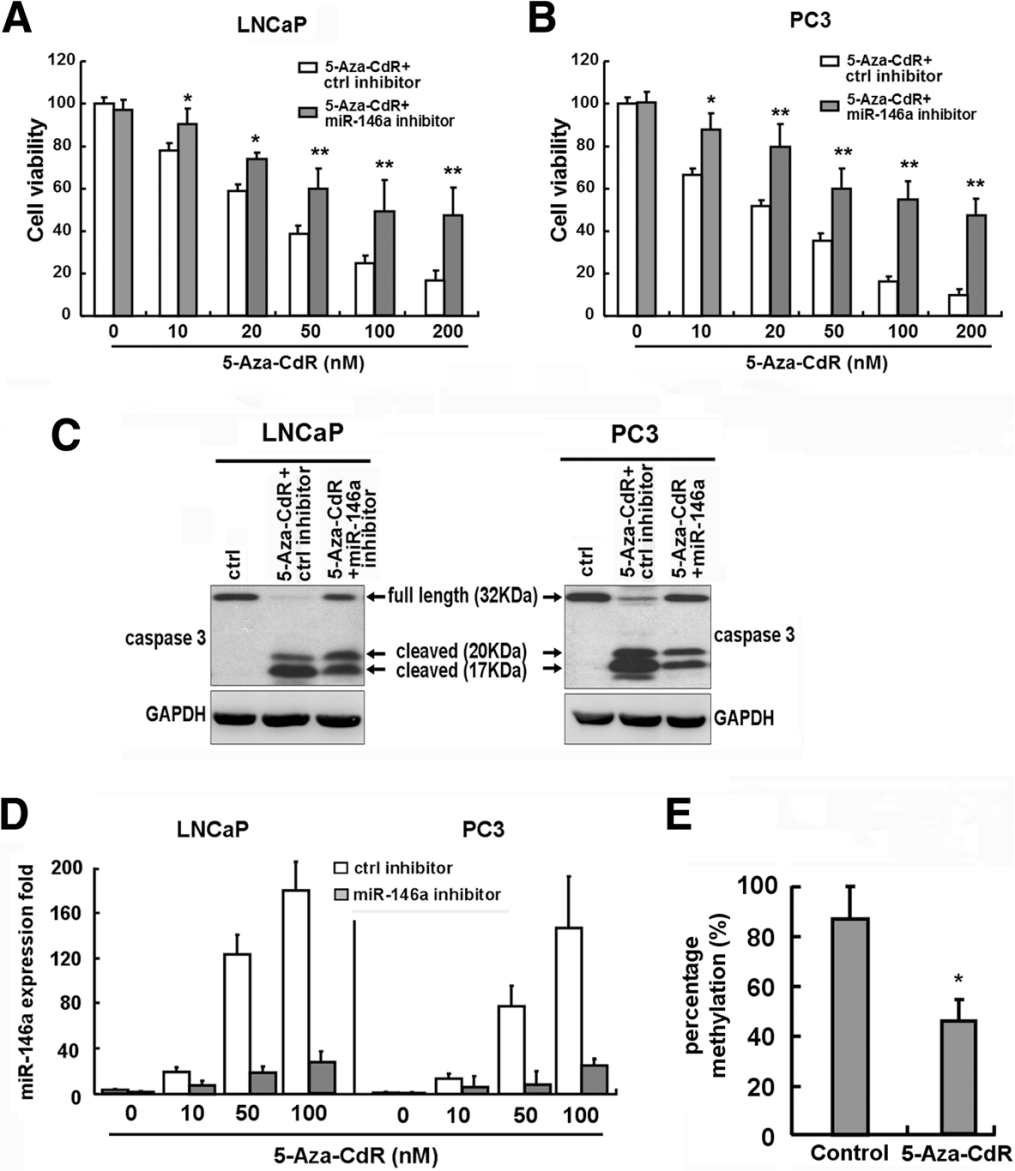
**The effect of 5-Aza-CdR and miR-146a inhibitors on the viability and apoptosis of LNCaP and PC3 prostate cancer cells.** LNCaP and PC3 prostate cancer cells transfected with the miR-146a inhibitor or control inhibitor were treated with increasing doses of 5-aza-CdR, replenished daily for up to 6 days. The relative cell viability was measured by the MTS assay **(A-B)** and apoptosis was detected by western blot analysis for the activation of caspase-3 **(C)**. MiR-146a expression was measured by qRT-PCR in LNCaP and PC3 cells treated with 5-Aza-CdR at various concentrations for 4 days transfected with control inhibitor or miR-146a inhibitor **(D)**. DNA methylation levels of miR-146a promoter region in 5-Aza-CdR-treated LNCaP cells by BSP assay **(E)**. The data represent the mean ± SD of triplicate wells from at least two experiments. *P < 0.05 and **P < 0.01 compared to the control.

We firstly examined the effects of 5-Aza-CdR on the viability of the LNCaP and PC3 cell lines using an MTS analysis. As shown in Figure 1A and B, treatment with 10 to 200 nM 5-Aza-CdR for 6 days induced cell death in a dose-dependent manner in both cell lines; 20 nM, 50 nM, and 100 nM 5-Aza-CdR caused 40%, 60%, and 75% reductions, respectively, in cell viability (as shown by white bars). To determine whether 5-Aza-CdR could effectively inhibit prostate cancer cell growth by induction of apoptosis, we examined the activition of caspase-3 in LNCaP and PC3 cells. As shown in Figure 1C, 5-Aza-CdR treatment resulted in significant increases in cleavage of caspase-3, which indicated that the mitochondrial apoptotic pathway was activated. The results indicated that both androgen-dependent and androgen-independent prostate cancer cell lines were susceptible to 5-Aza-CdR cytotoxicity.

Recent studies have demonstrated that the aberrant expression of miRNAs is closely associated with the development, invasion, metastasis, and prognosis of prostate cancer. MiR-146a was demonstrated to act as a tumor-suppressor gene in prostate cancer. Thus, to clarify the potential mechanism of 5-Aza-CdR, we determined whether miR-146a played an important role in 5-Aza-CdR-induced apoptosis in prostate cancer cells. As shown in Figure 1D, there was a dosage-dependent induction of miR-146a expression in both LNCaP and PC3 cells when treated with 5-Aza-CdR (as shown by white bars), with the expression of miR-146a in untreated LNCaP cells twice that in untreated PC3 cells. To estimate whether the effect of 5-Aza-CdR treatment on miR-146a expression was associated with the demethylation of miR-146a promoter, we analyzed the methylation status of miR-146a promoter region upon 5-Aza-CdR administration to LNCaP cells by BSP assay. As shown in Figure 1E, 5-Aza-CdR was downregulated twofold compared with the untreated cells. The result suggests that miR-146a expression was associated with its promoter methylation status.

To further confirm the association of increased miR-146a levels and 5-Aza-CdR-mediated apoptosis, the prostate cancer cells were transfected with miR-146a inhibitors and then treated with 5-Aza-CdR. We firstly examined the effects of knockdown of miR-146a in control and 5-Aza-CdR-treated cells. MiR-146a inhibitors significantly down-regulated the miR-146a expression level (*P* < 0.01) in both prostate cancer cells either untreated or treated by 5-Aza-CdR (Figure 1D) (as shown by gray bars). As shown in Figure 1A and B, miR-146a inhibitors significantly increased cell viability after 5-Aza-CdR treatment compared to the controls (as shown by gray bars). Additionally, treatment with miR-146a inhibitors significantly prevented 5-Aza-CdR-induced caspase-3 activation in both cell lines (Figure 1C), indicating that miR-146a played an important role in 5-Aza-CdR-induced cell death in prostate cancer cells, regardless of androgen dependency.

### 5-Aza-CdR increased the suppression of tumor growth in LNCaP xenograft model of castrated mice

**Figure 2:**
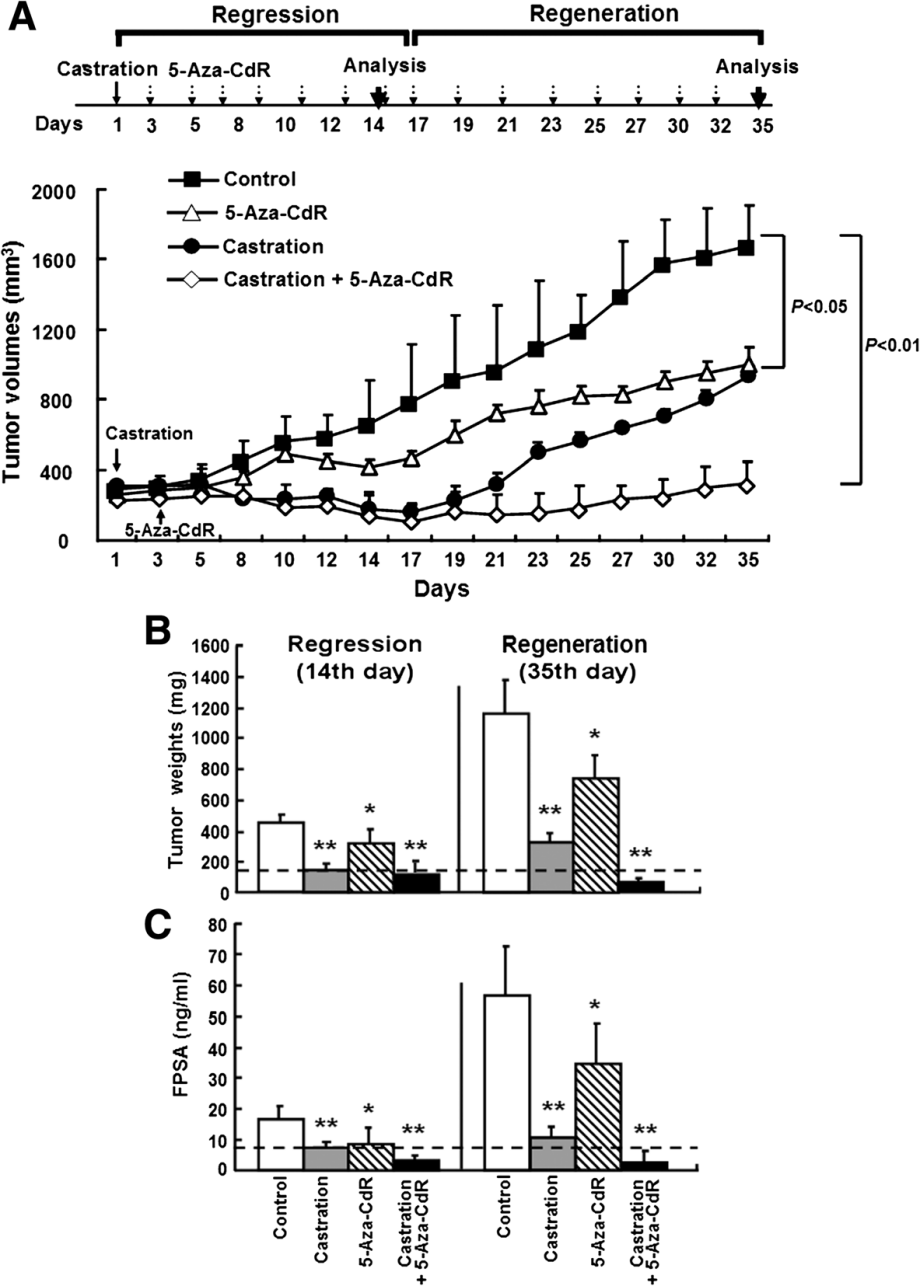
**Synergistic antitumor effect of the combination of 5-Aza-CdR and castration in vivo.** LNCaP cells were inoculated into the right thighs of BALB/c nude mice. When the tumor size reached to 250 mm^3^, the mice received an intraperitoneal injection of PBS (control), 5-Aza-CdR (0.25 mg/kg) thrice weekly, castration, or the combination of castration and 5-Aza-CdR (n = 12 per group). The tumor volume was monitored every two or three days **(A)**. The animals were euthanized on days 14 and 35 post-treatment (n = 6 per group at either time point) for DNA, RNA, and protein analyses. The tumor weights were determined **(B)**. PSA release into serum was measured by an ELISA analysis **(C)**. The data are the mean ± SD of one representative experiment. Similar results were obtained in three independent experiments. *P < 0.05, **P < 0.01 compared to the control mice.

To further investigate whether 5-Aza-CdR can delay prostate cancer progression, we establish subcutaneous LNCaP xenografts in a nude mice model. When the volume of xenografts reached to 250 mm^3^, the mice were randomly divided into 4 groups (n = 12) and treated with PBS as a control, 5-Aza-CdR, castration, or the combination of 5-Aza-CdR plus castration. After the 3rd day post-castration treatment, the tumors volumes began to decrease in both the castration treatment group and combination groups. On the 17th day of post-castration treatment, the tumor volumes in the castration group were reduced by 80%, to a much greater extent than in the control mice (Figure 2A); up to 17 days, tumor growth was considered in the regression stage. Thereafter, the tumors of the castration group began to grow uncontrollably. On the 35th day of post-castration treatment, the tumor size in the castration group was inhibited only by 45%; from 17 days to 35 days, the tumor growth was considered in the regeneration stage. The tumor growth in the combination group remained suppressed throughout this period, with the tumor size inhibited by 86% and 81% on the 17th and 35th days, respectively. In the 5-Aza-CdR treatment group, the tumors began to significantly grow more slowly than in the control group from the 10th day of post-treatment. On the 35th day of post-treatment, the tumor size in the 5-Aza-CdR group was inhibited by 41% compared to the control group.

The animals of the four groups were euthanized on the 14th and 30th days after castration, and the xenografts tissues were removed for the first analysis (n = 6) and the second analysis (n = 6), respectively. The tumor weights in the combination group were significantly lower than that treated with either therapy alone (*P* < 0.05) and the control group (*P* < 0.01) at the second analysis (Figure 2B).

To further assess the effects of 5-Aza-CdR on the transition of prostate cancer, we examined the free prostate-specific antigen (FPSA) concentrations, an important clinical parameter for screening and testing prostate cancer [20]. The results showed that the trend of FPSA concentration in the combination treatment was the lowest among the different groups in both analyses, similar to the tumor sizes and weights (Figure 2C). These data demonstrated that combination of 5-Aza-CdR and castration effectively suppressed the regeneration of castration-resistant prostate cancer in a nude mice model.

### MiR-146a expression was associated with tumor growth in the prostate cancer xenograft mouse model

**Figure 3:**
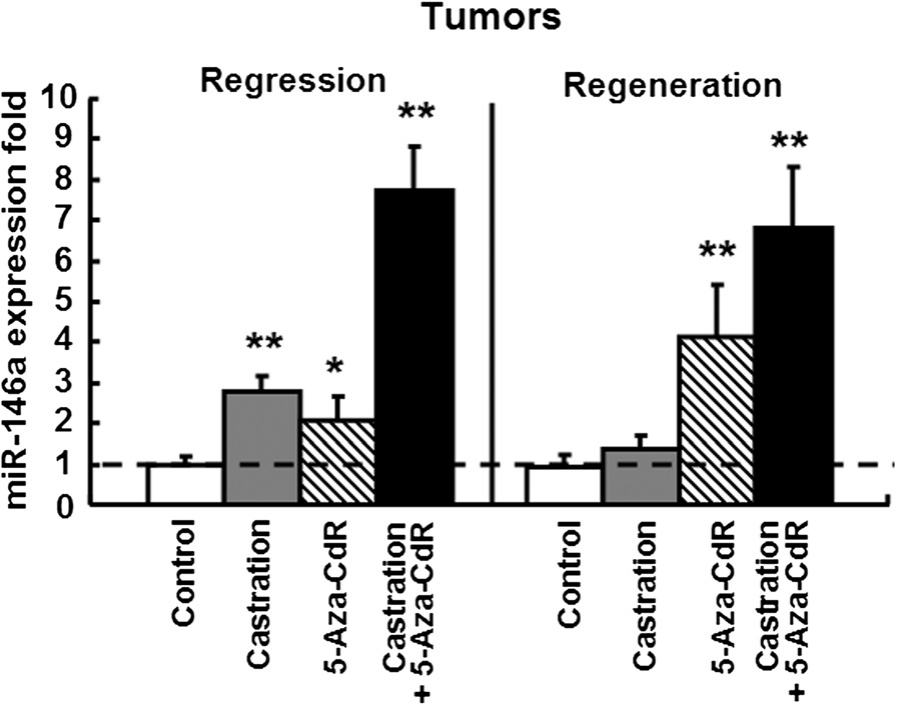
**miR-146a expression in human prostate cancer xenograft mouse models.** The miR-146a level was detected using qRT-PCR and described as the fold-change after normalization to U6 RNA in the tumor tissues of different mice groups (n = 6). The tumor tissue from the control mice of the first analysis was used to the calibrator samples. The data are the mean ± SD of one representative experiment. Similar results were obtained in three independent experiments. *P < 0.05, **P < 0.01 compared to the control samples.

MiR-146a expression in the xenograft tissues was detected by a qRT-PCR analysis. As shown in Figure 3, miR-146a levels in the 5-Aza-CdR-treated tumor tissue were significantly increased by approximately 2-fold at the first analysis and 4-fold at the second analysis compared to the controls, indicating that 5-Aza-CdR could effectively induce miR-146a expression in the prostate cancer xenograft mouse model. In the tumor tissue of the castrated mice, the miR-146a levels were also significantly increased by 3-fold in the regression stage but no different in the regeneration stage compared to the control. MiR-146a expression was at the highest levels in the combination group tissues, 7-fold at the former stage and 6-fold at the latter stage compared to the control mice, indicating a combined effect of 5-Aza-CdR and castration on miR-146a expression. The results suggested that miR-146a expression was negatively correlated with tumor growth in prostate cancer xenografts.

### The global DNA methylation levels were different among the different groups

**Figure 4:**
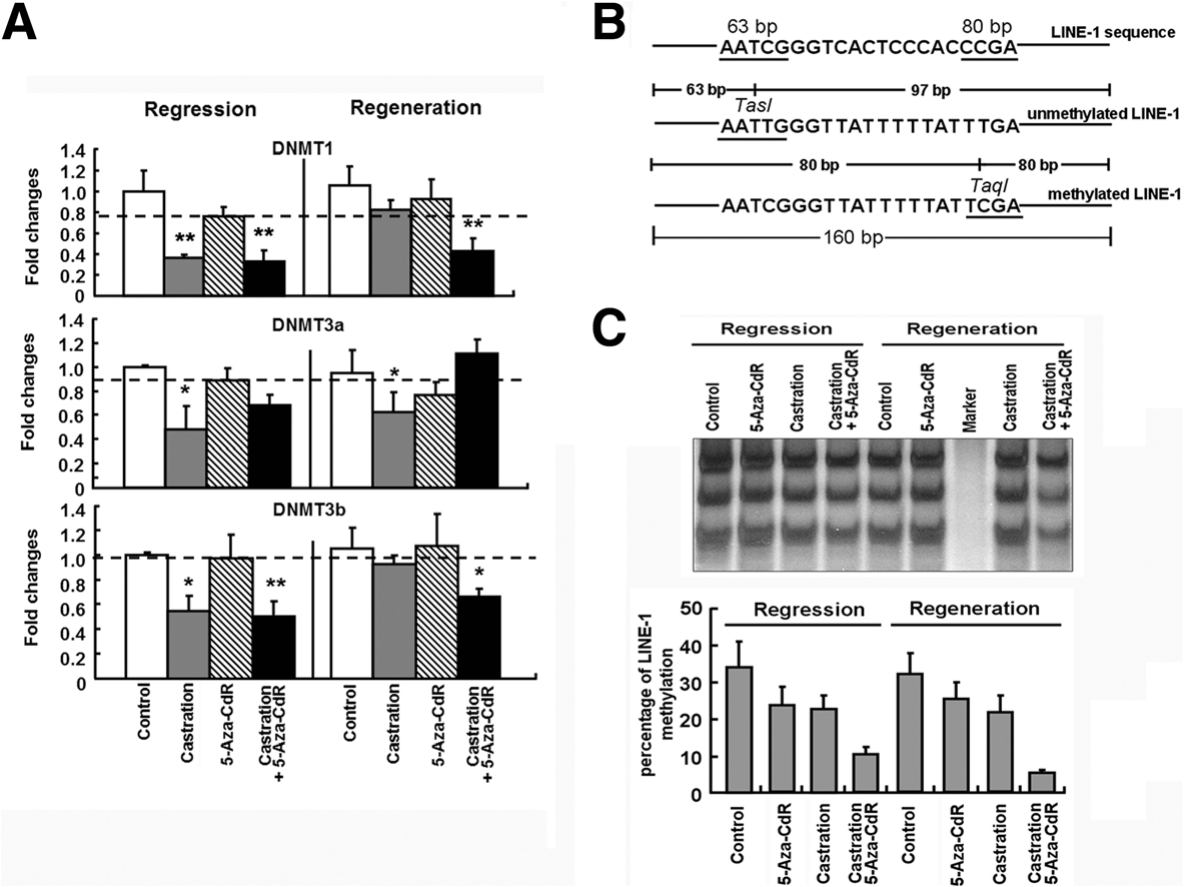
**The combination of 5-Aza-CdR and castration decreased the global gene methylation levels in human prostate cancer xenograft mouse models. (A)** The expression of DNMT1, 3a and 3b was measured by qRT-PCR and normalized to the expression of GAPDH in each sample of mice (n = 6). **(B)** A schematic illustration of COBRA LINE-1. DNA was extracted from the xenograft tissues, treated with bisulfite, and subjected to PCR. LINE-1 methylation level was assessed by *TasI-TaqI* double digestion within the 160-bp amplicon. The methylated amplicons (*TaqI* positive) yielded two 80-bp DNA fragments, whereas the unmethylated amplicons (*TasI* positive) yielded 63- and 97-bp fragments. **(C)** COBRA LINE-1 of xenograft tissues. The percentage of methylation is listed above each test. The data are the mean ± SD of one representative experiment. Similar results were obtained in three independent experiments. *P < 0.05 and **P < 0.01 compared to the controls.

To determine whether the treatments of 5-Aza-CdR and castration affected the global DNA methylation levels, we first detected DNMTs expression in the tumor tissues dissected from the xenografts to assay the global DNA methylation levels. As shown in Figure 4A, in the tissues of the first analysis, the expression of DNMTs was all slightly decreased in the 5-Aza-CdR-treatment groups (P > 0.05), whereas they were all significantly decreased in both the castration alone (P < 0.01) and combination group (P < 0.01). At the second analysis, the DNMT levels were mostly unchanged in the 5-Aza-CdR-treated mice (P > 0.05). In contrast, DNMT3a was decreased in the castrated mice (P < 0.05), and DNMT1 and DNMT3b were both significantly decreased in the combination group (P < 0.01 and P < 0.05, respectively), which was not consistent with the tumor sizes in the different groups.

To further confirm the global DNA methylation levels, we analyzed the DNA methylation of LINE-1, a surrogate marker of genome-wide methylation changes, by COBRA-IRS [21]. An outline and the principal of COBRA LINE-1 are illustrated in Figure 4B. The levels of LINE-1 methylation were significantly different among the different groups, highest in the control groups (35%) and lowest in the combined group either at the first analysis (10%), at the second analysis (5%) (Figure 4C). These results indicate that DNA methylation might be involved in the regulation of some critical gene expression in the progression of castration-resistant prostate cancer.

### The methylation levels of the miR-146a promoter were negatively associated with miR-146a expression in the different groups

**Figure 5:**
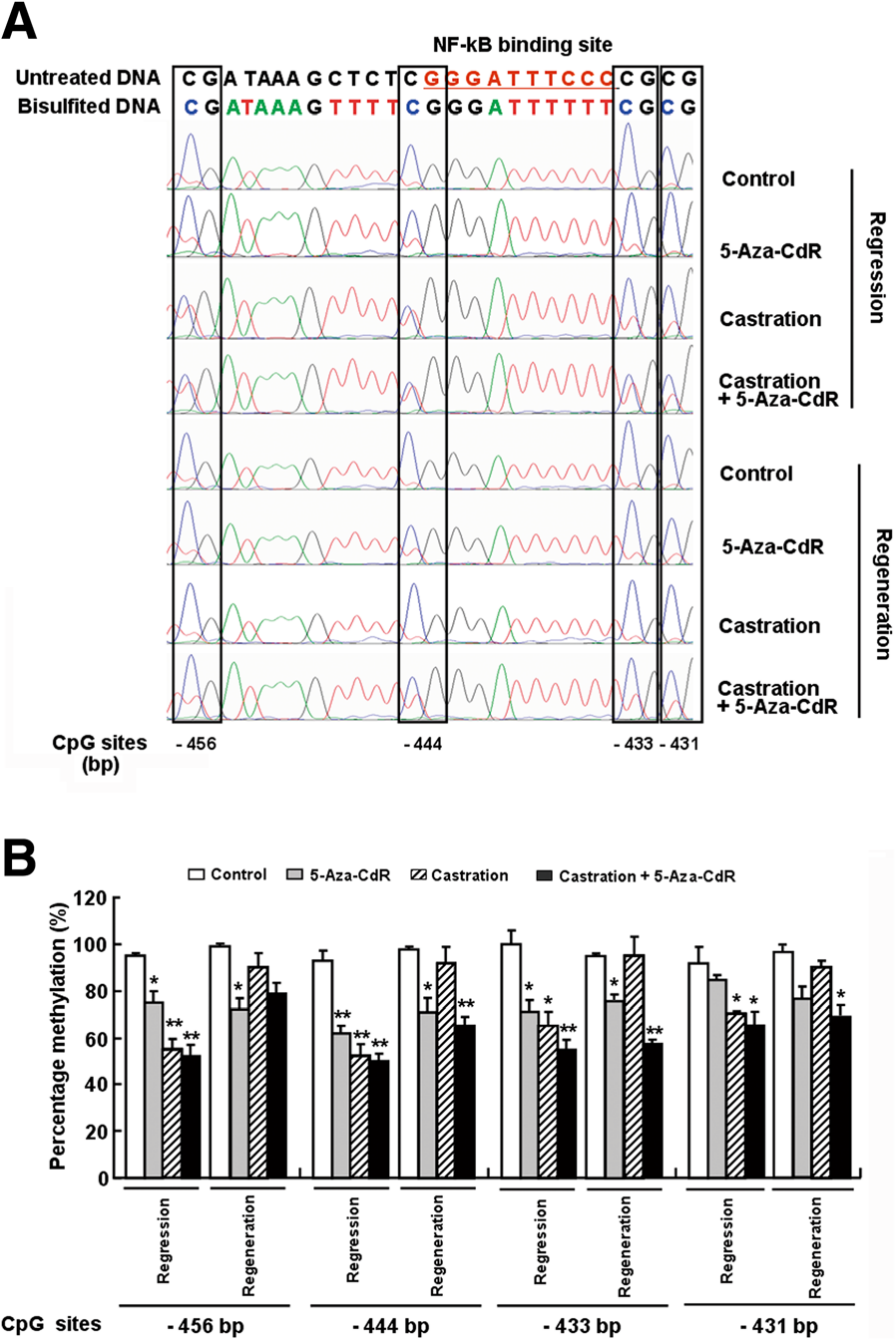
**Comparing the methylation levels of four CpG sites located in the miR-146a promoter collected from samples from the different group by direct BSP sequencing. (A)** Representative genomic sequencing chromatograms of the miR-146a promoter for each group. DNA was first treated with sodium bisulfite, and the amplified PCR products were then directly subjected to sequencing. The upper sequence is the untreated miR-146a promoter sequence; the lower sequences are treated sequences in different groups. The open boxes indicate the CpG sites. **(B)** Percentages of the methylation of four CpG sites in the miR-146a promoter. The methylation percentage of the individual CpG sites was calculated by the peak height of methylated residues (cytosine, C) divided by the sum of methylated and unmethylated residues (thymine, T). The methylation rate (%) is represented as the average ratio of methylated cytosines to total cytosines (methylated plus unmethylated) of all samples in each group. The data are the mean ± SD, n = 4–6 mice per group. *P < 0.05 and **P < 0.01 compared to the controls.

To determine whether DNA methylation was involved in the regulation of miR-146a expression, we analyzed the methylation of miR-146a promoter region of the different groups by the BSP assay. DNA was amplified using BSP primers, and sequencing chromatograms for the four CpG sites near and at the NF-κB binding site were shown in Figure 5A. The 5-Aza-CdR treatment group exhibited lower methylation levels at four CpG sites within the miR-146a promoter in comparison to the controls. The castration treatment and the combination treatment in the first analysis both showed the lowest methylation levels (50%) among the groups. However, the methylation levels increased to 90% in the castration group at the second analysis, similar to the control group. The combination group also showed high methylation levels at all CpG sites at the second analysis, with the exception of −444 bp and −433 bp, which were near the NF-κB binding site and is the key region involved in the transcriptional regulation of miR-146a [22,23] (Figure 5B). Overall, the methylation status of the the miR-146a promoter in different groups were similar to the levels of global DNA methylation (Figure 4C), and demethylation at −444 bp and −433 CpG sites were negatively associated with the expression of miR-146a, which may play a key role in miR-146a expression, while other CpG sites may play supportive roles in this regulatory mechanism.

## Discussion and conclusions

After prostate cancer patients undergo androgen deprivation as the first-line initial treatment, most patients develop to a more aggressive and androgen-independent status, with a median survival from 2 to 3 years. However, as the current clinical treatments for androgen-independent disease are not ideal, a therapy that either prevents or delays the development of AIPC is needed for these patients. In the present study, we found that 5-Aza-CdR could delay the emergence of lethal androgen-independent tumors in response to castration. Moreover, 5-Aza-CdR could augment miR-146a expression by decreasing the methylation levels of the miR-146a promoter in an LNCaP xenograft model of castrated mice, a result that was associated with the inhibition of prostate cancer progression. This is the first study to reveal potential molecular alterations by DNA methylation inhibitors as an effective therapy for preventing the relapse of castration-resistant prostate cancer.

The conversion to an androgen-independent phenotype is a complex process involving multiple molecular mechanisms. Prostate cancer cells can survive at low levels of androgens in castrated patients via androgen receptor (AR) mutation or amplification [24], or by increasing 5a-reductase activity to convert testosterone to dihydrotestosterone [25]. In addition, epigenetic alterations are the most common genome changes in prostate cancer cells and are associated with defects in gene function, contributing to carcinogenesis and helping the cancer cells survive and grow under androgen-independent conditions. Many specific genes are hypermethylated and inactivated during prostate cancer progression, including *APC, MDR1, GPX3*, and *p16*[26], and alterations in DNA methylation are now being used as molecular biomarkers for prostate cancer detection, diagnosis, and prognosis. 5-Aza-CdR, a DNMT inhibitor, can remove methyl residues from silenced genes, resulting in re-expression, and has been approved for use against hematopoietic malignancies. This inhibitor has also shown clinical efficacy in the treatment of metastatic lung carcinoma, acute lymphoid leukemia, chronic myeloid leukemia, and head and neck cancer. Furthermore, 5-Aza-CdR has been evaluated in hormone-independent metastatic prostate cancer patients in a phase II trial by Thibault *et al*. [27], and several studies have indicated the effect of 5-Aza-CdR on prostate cancer in animal models. Studies concerning combination chemotherapy of 5-Aza-CdR and chemotherapeutic agents have been performed, with the results suggesting the synergistic growth suppression of 5-Aza-CdR plus PTX in all PC cell lines [28]. In the present study, 5-Aza-CdR induced apoptosis of the androgen dependent (LNCaP) and androgen-independent (PC3) prostate cancer cells. The excessive expression of miR-146a and the demethylation of its promoter were exclusively found in both cell lines treated with 5-Aza-CdR, which was involved apoptosis of prostate carcinoma. Then we paid attention to if 5-Aza-CdR could delay the progression of prostate cancer to androgen independence in vivo.

Christoph *et al*. ever using a transgenic adenocarcinoma mouse model of prostate cancer demonstrated that the combined treatment of 5-Aza-CdR plus castration significantly prolonged survival and that 5-Aza-CdR appeared to delay the onset of androgen-independent disease [9]; however, that animal model did not mimic the course of the clinical disease. Nonetheless, the molecular mechanism by which 5-Aza-CdR inhibits the progression of prostate cancer remains unclear. Of the available human prostate cancer cell lines, only the LNCaP cell line is androgen responsive, PSA secreting, and immortalized in vitro. Thalman *et al*. demonstrated that the LNCaP progression model, unlike other human prostate cancer models, shares remarkable similarities with human prostate cancer and can transform a subpopulation to AI clones in response to acute or chronic androgen ablation. Based on previous studies, we utilized the LNCaP xenograft model to evaluate the effect of 5-Aza-CdR on the growth suppression of prostate cancer in castrated male mice. We observed that, within the initial 17 days, the xenograft tumor volumes were decreased in response to castration compared to the control, though the tumor volumes began to increase in the next stage, illustrating that the LNCaP xenograft model can mimic the course of the clinical states of the transition from initial androgen dependency to androgen independency, which is consistent with other reports [29,30]. 5-Aza-CdR treatment significantly suppressed tumor progression and delayed the regeneration of prostate cancer cells in the later stage compared to the castration treatment alone. Correspondingly, FPSA concentrations, an important clinical marker of prostate cancer progression, were also significantly down-regulated at the second stage in the combination treatment group compared to that in either the 5-Aza-CdR or castration alone groups. Although our study showed an effect of 5-Aza-CdR on the delay of the onset of androgen-independent disease in the mouse model, further research is necessary to evaluate whether 5-Aza-CdR can prolong survival and how long 5-Aza-CdR can delay the onset of androgen-independent disease in the castrated mouse model.

Additionally, we found that the expression of miR-146a was negatively correlated with tumor volumes, which significantly enhanced in the tumor xenografts of 5-Aza-CdR-treated mice and the androgen-dependent stage but not the androgen-independent stage of castrated mice, compared to control mice. In particular, the expression level of miR-146a was highest in the combined treatment (castration and 5-Aza-CdR), either androgen-dependent or androgen-independent stage. We further found the change trend of NF-κB activity, which is one of critical transcription factors for miR-146a expression, was consistent with miR-146a expression in tumor tissues of different groups (data not shown). Unfortunately, we could not reveal the potential mechanism involved in the regulation of NF-κB activity by 5-Aza-CdR or castration in the xenograft tumors of mice.

Since DNMT1, DNMT3A and DNMT3B are known to be involved in de novo methylation and the maintenance of methylation patterns of genes, we investigated the expression levels of all three members of the DNMT family. Our data clearly indicates that the DNMTs mRNA levels were all decreased after castration at the initial stage, yet were little changed at the later stage in different groups compared to the control in castrated mice. There was no a correlation between the levels of DNMT mRNA expression and the extent of global genomic DNA methylation, as measured by LINE-1 repetitive sequences. We deduced that the expression of DNMT family might have different effects on the pathogenesis of prostate cancer. A previous report demonstrated that DNMT3a and DNMT3b had overlapping functions in global remethylation during early embryogenesis [31]. However, the enzymes may have distinct cell- or tissue-specific functions during later embryogenesis or tumorigenesis [32]. In addition, DNMT family expressions could potentially be controlled at the post-transcriptional level in castrated mice. Lee JY *et al*. found that expression of DNMT3A and DNMT3B genes were post-transcriptionally regulated by several microRNAs, specifically *miR-1741, miR-16c,* and *miR-222,* and *miR-1632* via their 3’-UTR in cancerous ovaries of laying hens [33]. In this respect, more studies are needed to prove if the observed effect on DNMTs expression is specific for prostate cancer following castration.

Moreover, we found higher methylation of global genomic DNA at the later stage after castration versus the early stage, suggesting that the expression of some important anti-oncogenes was perhaps inhibited. However, 5-Aza-CdR treatment attenuated the methylation levels in the xenograft tumors of castrated mice at both stages. Among the different groups, the methylation levels of the miR-146a promoter were positively correlated with the global methylation status and negatively associated with miR-146a expression. It was notable that the miR-146a promoter methylation levels at the CpG site of −444 bp and −433 bp, in which an NF-κB binding site located, were lower than all the other CpG sites in the combination treatment mice at the second stage. NF-κB is one of critical transcription factors for miR-146a expression, so the methylation level of NF-κB binding region in miR-146a promoter methylation is a key regular mechanism involved in miR-146a transcription. Hypermethylation of the miR-146a promoter was associated with a loss of its expression in androgen-independent cells, and altered patterns of methylation in these cancer cells might represent a form of genome instability that has been hypothesized to occur during cancer progression. The loss of mir-146a was found in high-grade AIPC tissues but not in androgen-sensitive prostate epithelium, and miR-146a was demonstrated to be a tumor-suppressor gene in modulating HA/ROCK1-mediated tumorigenicity in androgen-dependent prostate cancer [12]. Our results suggest that the hypermethylation of the miR-146a promoter may be associated with aberrant miR-146a expression in vivo and possibly the resulting androgen insensitivity of these cells. However, to be fully understood, the precise mechanism of miR-146a action (its specific gene targets) in prostate cancer progression warrants investigation.

In conclusion, this is the first study to examine the effect of 5-Aza-CdR on the inhibition of prostate tumor growth in a castrated animal model. We also for the first time shed light on the molecular mechanisms underlying the synergistic action of 5-Aza-CdR and castration, and our findings suggest that the epigenetic regulation of miRNAs may play important roles in androgen-independent progression in patients after receiving androgen ablation therapy.

## Abbreviations

AIPC: Androgen-independent prostate cancer; 5-Aza-CdR: 5-Aza-2’-deoxycytidine; DNMT: A nucleoside analog inhibitor of DNA methyltransferase; FPSA: Free prostate-specific antigen; AR: Androgen receptor.

## Competing interests

The authors declare that they have no competing interests.

## Authors’ contributions

XW performed the experiments and wrote the manuscript. HG and LR carried out animal experiments and contributed to the evaluation of treatment effects. JG and YZ participated in analyzing the data. YZ, as the corresponding author, designed the protocol and made the draft of the manuscript. All authors read and approved the final manuscript.

## Pre-publication history

The pre-publication history for this paper can be accessed here:

http://www.biomedcentral.com/1471-2407/14/308/prepub
